# Meaning of life as a resource for coping with psychological crisis: Comparisons of suicidal and non-suicidal patients

**DOI:** 10.3389/fpsyg.2022.957782

**Published:** 2022-09-30

**Authors:** Olga Kalashnikova, Dmitry Leontiev, Elena Rasskazova, Olga Taranenko

**Affiliations:** ^1^Crisis Department, Eramishantsev Moscow City Clinical Hospital, Moscow, Russia; ^2^International Laboratory of Positive Psychology of Personality and Motivation, HSE University, Moscow, Russia

**Keywords:** meaning of life, psychological crisis, reasons for living, psychological resources, crisis of meaning, suicide

## Abstract

**Introduction:**

Meaning is an important psychological resource both in situations of accomplishment and in situations of ongoing adversity and psychological crisis. Meaning in life underlies the reasons for staying alive both in everyday and in critical circumstances, fulfilling a buffering function with respect to life adversities.

**Aim:**

The aim of the present study was to reveal the role of both meaningfulness, including specific sources of meaning and reasons for living, and meaninglessness (alienation) in patients suffering from profound crisis situations with or without suicidal intentions and behavior.

**Methods:**

The sample included 148 patients (all Caucasian) who were referred to a crisis center in Moscow, Russia. Seventy-seven patients (54 females, mean age 32.00 ± 11.98 years) reported a current crisis situation in their life but denied suicidal thoughts or behavior. Twenty-nine patients (21 females, mean age 31.55 ± 13.76 years) reported suicidal ideations but denied suicidal attempts or self-harming behavior. Forty-two patients (31 females, mean age 30.64 ± 11.38 years) had episodes of suicidal attempts or self-harming behavior accompanied by suicidal intentions. There were no significant gender or age differences between groups.

Participants completed a number of measures of different aspects of meaning and meaninglessness, well-being, ill-being and psychological resources. For some patients (*N* = 74), a clinical checklist was completed by their doctors assessing 28 various characteristics associated with the patient’s clinical status.

**Results and discussion:**

Meaningfulness and reasons for living were more helpful in distinguishing between reactions to profound crisis situations (suicidal intentions versus non-suicidal behavior) than were measures of well-being, ill-being, meaning crisis or personality resources. In both suicidal and non-suicidal crisis patients meaningfulness predicted more positive reasons for living. The relationship between meaningfulness and most reasons for living remained significant after controlling for clinically appraised suicidal “readiness,” acute stress and lack of social support. Self-transcendence was the major specific source of meaning predicting higher reasons for living after adjusting for general meaningfulness.

**Conclusion:**

The data cast some light on the psychological meaning of suicide. It follows that prevention efforts are to be focused not on eliminating the factors “pushing” one to suicidal behavior, but rather on supporting inner strengths conducive of a positive decision, *to be*, through enhancing meaningfulness and reasons for living.

## Introduction

Meaning in life is a double-edged psychological resource: it plays an important role both when one’s life is heading toward positive accomplishments and when, on the contrary, one is facing adversities, suffering, or psychological crises (see e.g., Baumeister and Vohs, 2002) which are also part of our everyday living. The focus of the present study is the role of life meaning as a buffer against the impact of crisis and trauma.

Nietzsche’s motto has become quite popular among contemporary students of meaning: “He who has a *why* to live for, can bear with almost any *how*” (quoted by Frankl, 1984, p. 84). Indeed, Viktor Frankl, the founder of logotherapy, stated that meaning was the critical resource for survival in inhumane circumstances, such as the Nazi concentration camps (Frankl, 1984). His argument was supported by evidence provided by numerous victims of war, natural disasters, imprisonment, or other adversities (e.g., Klinger, 1977; Eger, 2017).

Meaning (personal meaning) of life is a relational construct referring to the ties which connect our life to some superordinate context (see Baumeister, 1991a; Leontiev, 2013, 2017). Metaphorically, meaning is perhaps best characterized as a divine knot holding things together (Saint-Exupery de, 1979, p. 55). Meaningful life is thus coherent and conjoint, while meaningless life is fragmented and isolated (Leontiev, 2006). А growing number of studies define meaning as a fundamental human need (see a recent discussion in Martela et al., 2018) and as a resource which has a strong impact both on psychological and physical well-being (see e.g., Vail and Routledge, 2020).

Probably the most prominent effect of meaning is visible in extreme, highly challenging situations which may destroy people’s habitual activities, put into question their values and even their reasons for living. There are multiple studies, though the data are poorly systematized, on the role of meaning as a coping resource in times of psychological crisis, stress and trauma. In particular, Park described the model of meaning-making, noting that this process, if successful, leads to a better adaptation to the stressful event (Park, 2010). Her paper analyzed about 50 empirical studies focused on the changes in meaningfulness in certain stressful situations. Meaning-making, specifically finding situational meanings through the personal reappraisal of the traumatic situation, was also predictive of posttraumatic growth (Park and Ai, 2006).

Following are some more specific results, obtained mostly recently.

In a study of people after the collective trauma created by the 9/11 terrorist attacks, both searching for and finding meaning were important for successful adaptation (Updegraff et al., 2008, p. 718). People with more meaning searching activity were less likely to report any posttraumatic symptoms. Quite a number of studies deal with the COVID-19 pandemic. In particular, higher meaning in life was associated with a lower level of stress and anxiety caused by the pandemic (Trzebinski et al., 2020) and with a lower level of stress-induced mental disturbances (Schnell and Krampe, 2020). A coping strategy called the tendency to see Meaning in Negative Experiences (MINE) (Khei, 2019) was found to help in adapting to a stressful situation, not only in the moment, but in the long run (Yang et al., 2021). In another meta-analysis, a moderate negative relationship was found between meaningfulness as measured by Meaning in Life test (MIL; Steger et al., 2006) and Antonovsky’s Sense of Coherence (SOC) scale, on the one hand, and stress from confronting a cancer diagnosis, on the other. SOC showed an even stronger negative correlation with cancer stress (Winger et al., 2016).

With respect to the mechanisms by which meaning has a beneficial impact on coping with stressors, a two-way relationship has been established between meaningfulness and two coping patterns, *positive reinterpretation* and *proactive planning* (Ward et al., 2022). Specifically, meaningfulness “enhances people’s awareness about the broader purpose of their lives, it may facilitate recognition about the purpose and value of personal challenges, encouraging positive reinterpretation” (Ward et al., 2022, p. 3). The researchers note that they have not been able to demonstrate causal links between meaningfulness as measured by the MIL questionnaire and coping with diverse challenging events.

An important phenomenon is the non-opposite relationship between the positive and negative poles of life meaningfulness. Positive meaning, the feeling of meaningfulness and reasons for living seem to serve unambiguously as positive anti-stress buffers. However, the role of lack of life meaning, including specific negative meanings like the feeling of futility or feeling oneself to be rejected (Joiner, 2005), a crisis of meaning, or existential frustration (Frankl, 1969) is more ambivalent; for some people, or in some circumstances, lack of meaning may generate a positive urge toward the search for meaning. In a phenomenological study of the relationship between meaningfulness and meaninglessness (Debats et al., 1995), 122 respondents answered open questions about experiencing meaningfulness and meaninglessness. The researchers identified the following categories in their descriptions: meaningfulness, meaninglessness, no meaninglessness, and meaning as a way to cope with the crisis in the current situation. The category “no meaninglessness” was especially interesting, indicating that the structure, components and interrelations of the phenomena of meaningfulness and meaninglessness should not be considered as opposite poles of one dichotomy.

This phenomenon has long been discussed within the framework of existential analysis, but there has been no clear method for measuring it. An operationalization was proposed by Schnell and her colleagues. Schnell suggested that meaningfulness is not simply the opposite pole of a meaning of life crisis, but that these two concepts are more independent of each other than is commonly thought (Schnell, 2009). *Meaningfulness* and *crisis of meaning* comprise the two main scales of the Sources of Meaning inventory (SoMe; Schnell, 2009, see below). The independence of the two scales was tested and confirmed using correlation analysis, principal components analysis and confirmatory factor analysis: indeed, it is possible to have a combination of low meaningfulness with low crisis, a lack of awareness of the task of searching for meaning and a lack of desire to solve it. She called this pattern existential indifference (Schnell, 2010), comparing it to Frankl’s existential vacuum (Frankl, 1969) and Maslow’s metapathology or lack of Being-values (Maslow, 1976).

### Suicidal behavior as a reaction to psychological crisis: The buffering role of meaning and reasons for living

Suicide is the cause of many premature deaths in the contemporary world; the World Health Organization (WHO, 2014) estimates their number as over 800 thousand a year, and unsuccessful suicide attempts are impossible to count. It is an evidently unhealthy condition; at the same time suicide cannot be viewed purely as an illness or disease in the clinical meaning of the term. Contemporary data fail to provide specific causes of suicides, be it clinical symptoms or life challenges; the same objective obstacles or health issues sometimes result in a suicide attempt, and sometimes not. In the case of suicidal attempts, it is usually not easy to detect whether the failure of the attempt was due to external or internal barriers with respect to the person’s intention. No predictors have been reliably identified, and attempts to decrease mortality due to suicides have not been successful (Franklin et al., 2017). There are no reliable data which would allow treating suicide as an aspect of any clinical distortion or even reveal regular correlations of a suicide with any clinical syndrome (see a detailed discussion of this issue in Maung, 2021).

It is difficult to differentiate those who would make a suicide from those who would not before a suicidal attempt is made. The DSM-5 does not suggest a special category for suicide, stating that behavioral distortions like suicidal behavior and non-suicidal self-harming acts need additional investigation (Fehling and Selby, 2021). Likewise, in the ICD-11 suicidal behavior is not associated with any specific diagnosis.

Suicide is a multifactor, universally human phenomenon. Yet, every suicidal case is unique: in terms of its etiology, its biographical roots, its gender and age specificity and personal meaning contexts. No group, nation, or class of humans is free of suicidal cases. “Anyone can be at risk of suicide at any time” states the American Association of Suicidology,^1^ and the person at risk is the strongest resource in preventing suicide. No wonder that suicide has been a target of not only clinical but also social, moral, and theological discourse. In most cultures and religious confessions, it is treated as undesirable, morally wrong or sinful, though there are some exceptions, such as the Bushido, the Samurai code of conduct in medieval Japan.

### Psychological factors of reasons for living and suicide

The available data are more definite with respect to positive buffers against suicidal choice than with respect to the predictors of this choice. People who die from suicide are facing the same problems other people are facing; it is not the circumstances themselves, but rather their appraisal that causes emotional dysregulation (see e.g., Linehan, 1993; Turton et al., 2021). Moreover, suicidal impulses may emerge in the apparent absence of any life problems and obstacles, as for example seems to have been the case with Leo Tolstoy in the zenith of his fame (Tolstoy, 1983). Pain, hopelessness, despair, “psychalgia” (Shneidman, 1996) are regularly referred to as at least catalysts of suicidal behavior; however, a sudden and dramatic worsening of life obstacles, with a break in one’s expectations, seems to be more conductive to suicidal behavior than does long-term, ongoing misery (Baumeister, 1991b).

More interesting are the data on inner strengths (Seligman, 2002) which serve as buffers against destructive forms of behavior, including suicidal attempts. These inner strengths include, among others, positive emotions that make one’s life pleasant, as well as personality resources that make it more controllable, but most important seem to be positive meanings and reasons for living which provide the justification of the choice to live. “A person lives as long as he experiences his life as having meaning and value and as long as he has something to live for–meaningful projects that inspirit him and invite him to move into his future. …As soon as meaning, value and hope vanish from a person’s experience, he begins to stop living: he begins to die” (Jourard, 1971, p.93). The existing data suggest that meaning in life has a more important suicide preventive role than do the fear of suicidal impulses and religion-based cultural condemnation of suicide (e.g., Heisel and Flett, 2007; Wang et al., 2007).

Meaningfulness was studied as a buffer against suicidal behavior (Lew et al., 2020). It turned out that both presence of meaning in life and searching for meaning in life (MIL; Steger et al., 2006) negatively affected suicidal behavior, although the impact of the former was stronger. The search for meaning worked as a mediator between hopelessness and suicidal behaviors (Lew et al., 2020). It was also shown that both the presence of meaning and searching for it helped to reduce non-suicidal self-injury behavior (NSSI) (Conner et al., 2022).

In the context of suicide research, a highly relevant construct is that of reasons for living (RFL); indeed, the presence of such reasons is viewed as a buffer against acute experiences of depression, loneliness, loss and hopelessness (Linehan et al., 1983; Şahin et al., 1998). Reasons for living may be treated as a special case of meaning; however, we are not aware of specific studies that focus on relationships between these constructs. Meaning in life underlies the reasons for staying alive both in everyday and in crisis situations, fulfilling a buffering function with respect to life adversities. Reasons for living may prevent both suicidal ideation and suicidal attempts and yield a predictive value (Bakhiyi et al., 2016). Thus, RFL, together with meaning of life, is the target of our study. Though in some studies RFL has been considered as states (e.g., Demyttenaere et al., 2014), we treat them as trait variables. There are both theoretical and empirical reasons to consider life meaningfulness and positive reasons for living as the main and most universal precursors of a positive solution of the life/death dilemma.

### Suicidal ideation and suicidal actions

Distinguishing between suicidal ideation and suicidal behavior or combining them is still an open issue in the present-day empirical studies. Though most studies combine suicidal ideation with suicidal behavior (e.g., Posner et al., 2011; Franklin et al., 2017), in some studies we find a differentiated treatment of these two groups of phenomena (Klonsky and May, 2015; Klonsky et al., 2016). In particular, it has been established that the capability for suicide meaningfully distinguishes those who have attempted suicide (attempters) from those with suicidal desire but who have not attempted (ideators) (Klonsky et al., 2018). Another study concluded that individuals who attempt suicide have severe difficulties in problem solving, compared with those with mere suicide ideation and with psychiatric controls (Ghahramanlou-Holloway et al., 2012).

The distinction between suicidal ideation and suicidal action has been most pointedly articulated by Leslie Farber, who spoke of “the life of suicide, as distinguished from the act itself” (Farber, 1966, p.77). For Farber, such a life of suicide, that is, the awareness of this possibility as a part of the human condition, is not causally connected with making a suicide. “The awareness that it is possible for us to kill ourselves does not lead us to embrace suicide, any more than does the awareness that we are sinners prompt us to go forth and sin” (Farber, 1966, p.78). The mature person has enough personal and spiritual resources not to be enchanted by this possibility, but rather to reject it in favor of life. Suicidal thoughts are however socially stigmatized as something similar to suicidal attempts. This social disapproval rather than “the life of suicide” as such may be the reason why even the thought of suicide is often experienced as something pathological.

### The existentialist view on suicide: The role of meaning

In the middle of the 20th century existential philosophers (e.g., J.-P. Sartre, A. Camus, G. Marcel) added a new angle on viewing the problem. Considering the possibility of suicide meant for them ascending to a higher level of philosophical thinking, taking a conscious attitude toward one’s own life, making the latter an object of conscious choice, becoming “the master of one’s own death” (Camus, 1990, p. 336). Indeed, one of the key features of the existentialist approach to life is the acknowledgement of an opportunity to take a reflective and deliberate position toward one’s life and to deliberately change it. The problem of suicide is thus a critical issue for the existentialist worldview, and it cannot be analyzed without the consideration of its existential aspects. Indeed, suicide is a crossroad of all the four main existential challenges (Yalom, 1980): death, meaninglessness, freedom, and isolation. It suggests a higher level of relating to one’s own life, mastery over both life and death.

It is important to note that these authors referred to suicidal ideation, rather than suicidal actions. Suicidal ideation, that is emergence of thoughts about the possibility of ending one’s life, quite often is viewed in research context as indistinguishable from practical suicidal attempts, as a similar phenomenon. We think that they must be distinguished, and that suicidal ideation is not necessarily a negative phenomenon: it may more likely, on the contrary, bring the person to saying “no” to the option of suicide and “yes” to living, and such a person will value life more than ever, based on this conscious choice made in view of the available alternative to it. For some people, suicidal ideation can, we believe, serve as the basis for the intention to live. Exploring this proposition is one of the aims of the present paper.

### Suicidal ideation as wrestling with an existential challenge

We should take into consideration that a completed suicide is not so much an isolated action but rather a sad outcome of a more or less persistent dilemma, “to be or not to be,” in which life and death appear as two competing alternatives (see also Joiner, 2005; Joiner et al., 2009). Each of them has its own meaning; “Even a suicide believes in meaning – the meaning of dying, if not the meaning of further living. Otherwise he could not move a finger to fulfill his intention” (Frankl, 2005, p. 297). The point is which meaning will finally prevail – the meaning of living, or the meaning of giving up on living.

The idea of choosing between living and dying and the concept of a reflective “life of suicide” suggests that the person with suicidal ideations has not only urges toward a suicidal decision but also inner barriers against this decision, in favor of life. Emphasis on these positive barriers which serve as buffers against suicidal choice is in line with the message of positive psychology, stating that the royal road to mental health is supporting a person’s inner strengths which function as buffers against adversities, rather than trying to exclude negative influences. This resonates also with Meichenbaum’s (1985) idea of inoculation, according to which gradual exposure to small doses of a negative experience, rather than avoidance of the experience, better prepare the person to cope with stressful situations when they arise in the course of daily living. Kovacs and Beck (1977) also evaluated suicidal risks in terms of a competition between the wish to die and the wish to live.

As Victor Frankl (1984) noted in his well-known book of reflections as a Nazi concentration camp survivor, suicidal tendencies were less frequent in the camp than in everyday life. This tendency finds support in other authors (Bronisch, 1996), and indeed many explanations have been proposed (see e.g., Lester, 1997). The most plausible seems to be the explanation based on the meaninglessness of suicide under conditions in which only survival was a challenge but death was too likely and the chances for death mostly did not depend on the person’s preferences and efforts.

It is thus important to distinguish two aspects of the eventually suicidal path: (1) Becoming aware of the inevitability of death and the possibility of mastering it, and (2) Choosing in favor of either life or death (Leontiev, 2008). The first awareness seems to be associated with an advanced level of personality development, becoming able to be the master of one’s life and death in the context of realizing that one has two options from which to choose. “Until we can say no to life, we have not really said yes to it” (Hillman, 1964, pp. 63–64; see also Costello, 2019). This idea implies no suicidal risk, *per se*; on the contrary, it seems that those who have reflected much on this issue are less vulnerable to impulsive, self-destructive urges. The second aspect, the moment of choice, is the critical act that, for some, launches suicidal attempts.

## Empirical study of the role of meaning/meaninglessness in critical circumstances

It follows from the above considerations that meaning in life appears as an important factor to some degree predictive of the outcomes of profound life crises. Meaning is to be treated both in its positive aspects (meaningfulness as perceived presence of meaning in life being a buffer against worst outcomes) and negative aspects (crisis of meaning, meaninglessness, or alienation, as a precursor of psychological disturbances). The *aim* of the present study was to reveal the role of both meaningfulness, including specific sources of meaning and reasons for living, and meaninglessness (alienation) in perceived reasons for living in patients suffering from profound crisis situations with or without suicidal intentions and behavior.

We *hypothesized* that:

*Hypothesis 1 (H1)*: In patients suffering from profound crises without suicidal intentions, life meaningfulness, reasons for living, self-regulatory, or stress-buffering resources (hardiness, tolerance for ambiguity, action orientation, coping strategies) and well-being (subjective happiness, subjective vitality) are higher than in patients suffering from profound crises with suicidal ideations and suicidal behavior.

*Hypothesis 2 (H2)*: Both higher meaningfulness and lower crisis of meaning predict higher reasons for living in both groups. We also hypothesized that in patients with suicidal intentions and behavior these effects would be stronger, such that meaningfulness and lower crisis of meaning would better predict reasons for living in this group. As well, we predicted an interaction effect between meaningfulness and crisis of meaning, such that crisis of meaning would have a more negative effect on reasons for living in those with lower meaningfulness

*Hypothesis 3 (H3)*: The effects of meaningfulness, crisis of meaning and, probably, specific sources of meaning on reasons for living remain stable after adjusting for clinical appraisals of patients.

## Materials and methods

### Sample

Data were collected through 2010–2016 from residents of the special clinics of crisis care, Crisis Department №2 of Eramishantsev Moscow City Clinical Hospital. This department was established for patients voluntarily requesting help because of their psychological crisis or difficult life obstacles, including thinking about suicide or suicidal attempts. Most of the patients are without manifest psychiatric diagnoses; those with acute psychoses, serious alcohol or drug addictions and major somatic or neurological diseases are typically not admitted. Data collection was conducted in the middle of their inpatient treatment period which lasted about 1 month, in the clinic. We excluded from the sample patients with organic and psychotic disorders–18 cases in total.

The initial sample included 148 inpatients 18–65 years old., 105 of them females. Of them 75, or 49.3% had completed university level education and 24 (15.8%) had started but not completed university, 32 (21.1%) had completed high school level; 2 (1.3%) reported middle school level, and 19 (12.5%) gave no education data. Diagnoses by ICD-10 included: F20-28 – 23 participants (Schizophrenia, schizotypal and delusional disorders); F31-34 – 17 participants (Mood [affective] disorders); F40-48 – 83 participants (Neurotic, stress-related and somatoform disorders); F50 – 1 participant (Behavioral syndromes associated with physiological disturbances and physical factors); F60-69–24 participants (Disorders of personality and behavior in adult persons). We considered mixed diagnosis sample acceptable, because in the domain of suicide research and treatment transdiagnostic approach focusing on behavior rather than clinical diagnosis is often applied; there have been attempts to include suicidal behavior disorder as a separate DSM-5 entity (Oquendo and Baca-Garcia, 2014).

Patients were classified into three groups *post-hoc*, by an expert psychologist who was not directly involved in treating the patient, based on their final medical history (official medical records after the end of treatment). These records contained psychiatric anamnesis, concomitant diagnoses, biographical information, admission obstacles, cases of suicidal affects, ideations, or actions, the character and the result of treatment.

Seventy-seven patients (54 females, mean age 32.00 ± 11.98 years old) reported a current crisis situation in their life but denied suicidal thoughts or behavior (group NO). Twenty-nine patients (21 females, mean age 31.55 ± 13.76 years old) reported suicidal ideations but denied suicidal attempts or self-harming behavior (group ID). Forty-two patients (31 females, mean age 30.64 ± 11.38 years old) had experience of suicidal attempts or self-harming behavior accompanied by suicidal intentions (group BE). It should be noted that self-harming behavior is considered in the clinic only in case of prominent suicidal behavior. Minimal and non-suicidal injuries are typically not admitted to the clinic and are not included in the diagnostic descriptions of behavior. Consequently, we treated both patients with suicidal attempts or self-harming behavior as the same group.

There were no significant gender and age differences found between groups. The differences on most of the additional measures (see below) were also insignificant, including subjective happiness, subjective vitality, anxiety, depression, hardiness, cognitive insight, ways of coping, action control, subjective alienation, and tolerance for ambiguity. On the contrary, significant differences between the groups were found on sources of meaning and reasons for living (see below in the results section).

All the patients in the course of an individual session with the psychologist were asked to complete a battery of inventories printed on paper; prior to completion, an informed consent was obtained.^2^ The participation was voluntary; no incentives were offered. There were no cases of refusal to participate in the investigation; indeed, the patients perceived it as a part of the whole treatment process. Surveys were completed individually, without assistance or interference from the psychologist; it took them on average about 2 h to complete the materials. The measures used in the study were divided into two groups: those assessing the main targets of the study (meaning, alienation, reasons for living) and several additional ones checking for differences in personality resources and emotional states and symptoms. The additional measures yielded no significant differences between the groups.

The survey data reflected the patients’ current situation which typically did not change much after getting to the clinic. We did not consider the impact of therapy or other interventions.

### Main measures

(Cronbach’s alphas are presented in Table 1):

1. *The reasons for living inventory (RFL)* (Linehan et al., 1983; Russian version by Olina, 2010). The RFL was developed as a tool for researching the motives that serve as a buffer to prevent suicide attempts. As a result of factor analysis, six groups of reasons covered the explanations why people choose life rather than death: (1) need to cope with problems; (2) responsibility for the family; (3) motives associated with children; (4) fear of suicide; (5) fear of social disapproval; (6) moral attitudes that prevent the making of suicide.
2. *The sources of meaning and meaning in life questionnaire (SoMe)* (Schnell, 2009). The full version of the questionnaire includes 151 items. Two main SoMe scales are meaningfulness and crisis of meaning. Other items make 26 subscales reflecting different sources of meaning; each of them includes from 3 to 6 questions. These subscales are organized in 5 secondary scales: Horizontal self-transcendence (commitment to objectives beyond one’s immediate needs); Vertical self-transcendence (orientation toward an immaterial, cosmic power); Self-actualization (employing, challenging, and fostering one’s capacities); Sense of order (holding on to traditional values and morality, practicality, decency); Well-being of themselves and those around them (cultivating and enjoying life’s pleasures in privacy and company). A Russian validation (Bolotova and Leontiev, 2016) which retained all 151 items confirmed the validity of two general and 5 secondary sources scales, but not of the 26 primary sources, which is why the latter were not used.
3. *Noetic orientations test (NOT)* (Leontiev, 1992) is a modification of the “Purpose in Life Test” (PIL) (Crumbaugh and Maholick, 1964). It includes 20 polar Likert-type statements with seven gradations for the answers. Its main scale (subscales were not used in this study) is a measure of life meaningfulness.
4. *Subjective alienation inventory* (Osin, 2007) was created on the basis of the alienation test (Maddi et al., 1979). It includes 60 items and measures four forms of alienation: vegetativeness, powerlessness, nihilism and adventurousness. These forms of alienation can be expressed in different areas of life: work, society, other people (close relationships), family and the person him/herself. Answers to questions are given as a percentage from 0 to 100%.

**Table 1:** Reasons for living, meaningfulness, meaning sources and clinical appraisals in the three groups of patients.

	Suicidal attempts or self-harming behavior (BE)		Suicidal ideation (ID)		No suicidal thoughts or behavior (NO)		Fisher’s F	Effect size η^2^	Cronbach’s alpha
	Mean	St. dev.	Mean	St. dev.	Mean	St. dev.			
RFL–Coping with Problems	81.70^a^	28.67	86.35^b^	29.28	106.12^a, b^	23.90	11.14^**^	0.13	0.95
RFL–Family Responsibility	29.46^a^	10.56	28.95	8.30	34.43^a^	7.68	5.06^**^	0.07	0.87
RFL–Relationship with Children	12.56	5.72	11.63	5.53	14.60	4.86	3.17^*^	0.04	0.87
RFL–Fear of suicide	21.48	9.51	23.57	8.82	20.75	8.13	0.77	0.01	0.76
RFL–Fear of social disaproval	9.22	4.67	8.74	4.60	10.60	3.79	2.04	0.03	0.73
RFL–Moral Reasons	11.44	7.08	11.26	6.22	14.37	5.87	3.25^*^	0.04	0.79
NOT - Meaningfulness	78.21	22.05	74.21	17.66	83.71	22.01	2.32	0.03	0.86
Alienation (vegetativeness)	41,19	15,35	45,54	15,24	41,45	16,16	0.818	0.01	0.94
SoMe–Meaningfulness	11.77^a^	6.43	14.83	6.19	15.88^a^	5.51	6.63^**^	0.08	0.61
SoMe–Crisis of meaning	12.98	7.60	14.10	5.83	11.53	7.02	1.60	0.02	0.75
SoMe–Vertical self-transcendence	21.49	7.92	22.86	6.37	23.99	8.36	1.38	0.02	0.80
SoMe–Horizontal self-transcendence	71.75^a^	19.49	81.57	18.51	83.22^a^	19.47	4.98^**^	0.06	0.89
SoMe–Self-transcendence–Total score	93.24^a^	24.43	104.43	22.53	107.20^a^	25.58	4.44^*^	0.06	0.81
SoMe–Self-actualization	137.43	37.10	145.22	31.42	140.30	31.44	0.47	0.01	0.85
SoMe–The sense of order	75.68^a^	19.17	79.17	16.83	84.41^a^	12.94	4.43^*^	0.06	0.81
SoMe–Well-being of themselves and those around them	134.64	29.18	137.64	27.57	137.43	28.55	0.15	0.00	0.84
Suicidal readiness	2.52	0.42	1.82	0.95	1.84	0.85	5.44^**^	0.36	0.93
Self-injuries and deviant behavior	1.52	0.73	1.07	0.61	1.34	0.73	2.08	0.24	0.91
Affective symptoms	2.74	0.35	2.30	0.76	2.57	0.56	2.94	0.28	0.78
Social support importance and deficit	2.33	0.53	1.61	0.68	2.18	0.56	8.50^**^	0.44	0.76
Acute stress symptoms	2.49	0.70	2.29	0.61	2.51	0.68	0.75	0.14	0.69

^*^p < 0.05, ^**^p < 0.01. Values with the same superscript letter (^a, b^) significantly differ from each other by *post-hoc* Scheffe pairwise comparisons *p* < 0.05.

#### Additional measures

5. *Subjective Happiness Scale* (Lyubomirsky and Lepper, 1999; Russian version by Osin and Leontiev, 2020). The scale consists of 4 items, rated on a seven-point Likert-type scale.
6. *Two scales of subjective vitality* (Ryan and Frederick, 1997; Russian version by Aleksandrova, 2014): the scales of subjective vitality as a state (Vt-s) and subjective vitality as a personal disposition (Vt-d). Each of them consists of 7 items, rated on a seven-point Likert-type scale.
7. *The hospital anxiety and depression scale (HADS)* (Zigmond and Snaith, 1983). This scale measures the two most common forms of psychological disorders in medical patients with 7 questions for anxiety and 7 items for depression, scoring the intensity of the symptoms from 0 to 3.
8. *Hardiness survey* (Maddi and Khoshaba, 2001; Russian version by Leontiev and Rasskazova, 2006). Hardiness as the integrative variable predictive of enduring stresses without health impairment is composed of three dispositional components: commitment, control, and challenge. The actual version used in the present study includes 45 four-point Likert-type items (from 1 = No to 4 = Yes).
9. *The Beck Cognitive Insight Scale (BCIS)* (Beck et al., 2004; Russian version by Rasskazova and Pluzhnikov, 2013). The BCIS consists of 15 four-point Likert-type items which measure Self-reflectiveness (9 items) and Self-certainty (6 items).
10. *Ways of coping questionnaire* (Folkman and Lazarus, 1980; Russian version by Kryukova and Kuftyak, 2007). The scale includes 50 four-point Likert-type items which assess 8 different types of coping: confrontation, self-distance, self-control, search for social support, taking responsibility, escaping, solution planning, and positive reappraisal.
11. *Action Control Scale* (Kuhl, 1994); Russian version by Wassiljev et al. (2011) the questionnaire consists of 36 items containing polar statements. The items are grouped into three scales: control over the action during planning, control over the action during the implementation, control over the action in case of failure.
12. *Tolerance for ambiguity* (McLain, 1993; Russian version by Leontiev et al., 2016). The scale consists of 22 seven-point Likert-type items.

### Clinical appraisals

In order to evaluate the clinical condition of patients, a clinical checklist including 28 various characteristics was appraised by clinicians for each patient using 0–4 scale Likert scale. The structure of the checklist was elaborated by the first author together with the Head of the Federal Suicidological Center Vladimir Voitsekh and included the manifestations of self-destructive and deviant behavior (suicidal thoughts, actions, attempts, suicidal motives, episodes of alcoholism and drug abuse etc.), characteristics of emotional, cognitive, value, behavioral aspects (accentuations, self-blame, feeling of loneliness, depression, anhedonia, anger, perfectionism, rigidity of thinking, the importance of stress, feeling of social support, manifestations of religiosity etc.). Twenty-eight clinical characteristics were formulated for five domains (descriptive statistics and Cronbach’s alphas are presented in Table 1): suicidal readiness (e.g., “Suicide is a protest”, or “Suicide is a refusal reaction”), self- injuries and deviant behavior (e.g., “The desire to get a tattoo”, or “Episodes of drug abuse”), affective symptoms (e.g., “Presence of selfincrimination ideas at the moment”, or “Depression in the most recent period of time”), social support importance and deficit (e.g., “Dysfunctional (destructive) elements in the family”, or “Deficit of communicability”), acute stress symptoms (e.g., “Personal stress”, or “Psychalgia”).

For technical reasons, full checklist data were available only for a subsample of the initial sample including 74 patients (35 without and 39 with suicidal intentions, 21 males and 53 females, mean age 31.49 ± 12.14 years). Due to smaller sample size for clinical appraisals we added them into separate analysis after the major part of results including the whole sample. There were no significant gender or age differences between the subsample and the initial sample.

### Data processing

Data were processed in SPSS Statistics 23.0. Descriptive statistics and Cronbach’s alphas are presented in Table 1. One-way ANOVA (with Scheffe’s pairwise comparisons) was used to reveal differences between 3 groups of patients. In order to test H1 and H2, moderation analysis using regression was performed separately with each of the six reasons for living and their composite indexes as the outcome variables. At the first step, independent variables included dummy-coded indicators of the Groups BE and NO (group ID was the reference one). At the second step, we added centered variables of meaningfulness and meaning crisis. The third step included moderators computed by multiplying each group indicator with meaningfulness and meaning crisis. Finally, at step four we added into the equation the composite describing the interaction between meaningfulness and meaninglessness and three-level interactions of this composite with groups. We did not include age and gender as covariates while there were no relationships between them and reasons for life.

To reveal whether not only meaningfulness and crisis of meaning but also specific sources of meaning (e.g., its content) were important for RFL all the moderation analyses were repeated. As before, step 1 included dummy-coded group membership and step 2 included centered variables of crisis of meaning and meaningfulness as well as their interactions with group variables. However, unlike in the previous section, we used at step 2 stepwise analysis to reduce the number of variables that were unrelated to RFL. Step 3 included centered sources of meaning (horizontal and vertical self-transcendence, order, self-actualization and well-being). At step 4 we added their interactions with the dummy-coded group variables. At both steps only variables that predicted the dependent variable at least in one group remained in the equation. Thus, improvement of the model at step 3 indicated that there were sources of meaning that predicted RFL independently of general meaningfulness and meaning crisis. Improvement at step 4 indicated that there were meaning sources which were related to RFL only in some groups.

To reveal the relationships between clinical characteristics and RFL in the three groups of patients a series of seven separate moderation analyses (for each RFL) was performed. At the first step we added dummy-coded variables to reflect clinical group membership (the group ID was the reference group) and all five clinical characteristics.

## Results

### Psychological resources and well-being in people suffering from profound crisis situations with or without suicidal intentions

The three groups of patients differed by four out of six scales of RFL Questionnaire (excluding Fear of Suicides scale and Fear of Social Disapproval scale) (Table 1). In general, readiness to live for moral reasons, responsibility for family, relationship with children and to cope with problems were the highest in the NO group and the lowest in the BE group. Similarly, three out of seven main scales of the Sources of Meaning Questionnaire (Meaningfulness, Horizontal Self-Transcendence, Order) differed across the groups: they were the highest in the NO group and the lowest in the BE group. Meaningfulness as measured by the NOT revealed the same pattern.

The groups did not significantly differ from each other (*p* > 0.15) on happiness, vitality, anxiety, depression, hardiness, cognitive insight, coping strategies, action control, subjective alienation, or tolerance to ambiguity.

In line with Schnell’s hypothesis, meaningfulness and the crisis of meaning appeared as related but different constructs (*r* = −0.57, *p* < 0.05 in the NO group, *r* = −0.57, *p* < 0.05 in the ID group, *r* = −0.21, *p* > 0.10 in the BE group). Moreover, in the BE group this relationship was less prominent than in the NO group (*p* < 0.05) and marginally less prominent than in the group ID (*p* < 0.10).

### Meaningfulness and crisis of meaning as predictors of reasons for living

According to moderation analysis, for all but one dependent variable only the first and the second steps reached significant changes in *R*^2^ (Table 2). The coping with problems reason, the responsibility for family and children reasons, and the moral reason were higher in the NO group as compared to the other two groups which did not significantly differ from each other. All the patients with higher meaningfulness reported more RFL while higher crisis of meaning after adjusting on meaningfulness predicted lower scores on the coping with problems reason. Thus, the effect of crisis of meaning differed from the mere inverted effect of meaningfulness only with respect to the coping with problems reason.

**Table 2:** Meaningfulness and crisis of meaning as predictors of reasons for living: results of hierarchical regression.

Dependent variables		Step 1		Step 2		Step 2–verification	
		BE group	NO group	Meaning-fulness (SoMe)	Crisis of meaning (SoMe)	Meaningfulness (NOT)	Alienation (vegetativeness)
RFL–Coping with problems	*β*	−0.07	0.34^**^	0.40^**^	−0.29^**^	0.48^**^	−0.04
	Δ*R*^2^	15.8%^**^		31.6%^**^		24.9%^**^	
RFL–Responsibility for family	*β*	0.03	0.30*	0.29^**^	−0.07	0.13	−0.11
	Δ*R*^2^	8.0%^**^		9.7%^**^		4.9%^*^	
RFL–Relationships with children	*β*	0.08	0.28^*^	0.47^**^	−0.11	0.38^**^	0.05
	Δ*R*^2^	5.1%T		25.4%^**^		11.3%^**^	
RFL–Moral	*β*	0.01	0.24 T	0.20 T	−0.10	0.16	0.03
	Δ*R*^2^	5.1%T		6.4%^*^		1.9%	

*T*–*p* < 0.10;

* *p* < 0.05;

** *p* < 0.01.

There were significant moderation effects between meaningfulness and meaninglessness regarding moral RFL: the interaction between meaningfulness and crisis of meaning and its second order interactions with groups reached the level of significance *p* < 0.05 (*β* = −0.81, *p* < 0.01, *β* = 0.62, *p* < 0.05, *β* = 0.67, *p* < 0.01, respectively, Δ*R*^2^ = 7.4%, *p* < 0.05). Simple regressions demonstrated that in the BE group moral RFL were related neither to meaningfulness, nor to crisis of meaning nor to their interaction. In the NO group more moral reasons were associated with higher meaningfulness (*β* = 0.40, *p* < 0.01) while in the ID group the disbalance between meaningfulness and crisis of meaning (e.g., both are high or both low) was related to less moral RFL (*β* = 0.48, *p* < 0.05).

We repeated all the analyses using other variables of meaningfulness and meaninglessness: Meaningfulness by NOT and Alienation (Vegetativeness) as alternative measures. For coping with problems and relationships with children, we found the same positive relationship between meaning in life and reasons to live. However, we did not find three-level interactions for moral reasons to live.

In should be noted that for the coping with problems reason after adding interactions of meaningfulness by NOT and vegetativeness with groups of patients there was a clear negative relationship between vegetativeness and life for coping with problems (*β* = −0.55, *p* < 0.05). However, this relationship was stronger in the BE group (*β* = 0.42, *p* < 0.05) as compared to both other groups. In other words, alienation was a more important negative factor of the coping with problems reason in patients with self-harming behavior than in the ID and NO groups.

Fear of suicide was not predicted by group or meaningfulness but it was positively related to the crisis of meaning after adjusting for dummy-coded groups (*β* = 0.20, *p* < 0.05, Δ*R*^2^ = 3.8%, *p* < 0.05). Furthermore, fear of suicide was associated to alienation after adjusting for groups and meaningfulness by NOT (*β* = 0.33, *p* < 0.01, Δ*R*^2^ = 10.5%, *p* < 0.01). Fear of social disapproval was not predicted by group, meaningfulness or crisis of meaning.

### Sources of meaning as additional predictors of reasons for living

For all but one (fear of suicide) RFL a stepwise regression revealed sources of meaning that were associated to them after adjusting for dummy-coded groups, meaningfulness and meaninglessness (Table 3). Relationships with children and fear of social disapproval were higher in those with higher horizontal transcendence and lower self-actualization. The coping with problems reason was also higher in patients with higher horizontal self-transcendence while moral reasons were related to higher self-transcendence only in the NO group. In general, only fear of suicide and responsibility for family were not predicted by horizontal self-transcendence. However, responsibility for family was predicted by vertical self-transcendence. A similar pattern was found for moral reasons.

**Table 3:** The effects of sources of meaning on RFL in patients from different groups.

Dependent variables		Step 3				Step 4			
		SoMe–Horizontal self-transcendence	SoMe–Well-being	SoMe–Vertical self-transcendence	SoMe–Self-actualization	Interaction: BE group × Horizontal self-transcendence	Interaction: NO group × Horizontal self-transcendence	Interaction: BE group × Self-actualization	Interaction: NO group × Self-actualization
RFL–Coping with problems	Β	0.21^*^	–0.15 T	–	–	–	–	–	–
	Δ*R*^2^ (47.5%^**^)	3.1%^*^				–	–	–	–
RFL–Responsibility for the family	Β	–	–	–	–	–			
	Δ*R*^2^ (17.3%^**^)	11.7%^**^				–	–	–	–
RFL–Relationship with children	Β	0.27^*^	–	–	–	–			
	Δ*R*^2^ (32.2%^**^)	9.6%^**^				–	–	–	–
RFL–Fear of social disapproval	Β	0.36^**^	–	–	−0.30^*^	0.47^*^	0.62^*^	−0.86^**^	−0.72^**^
	Δ*R*^2^ (3.5%)	7.7%^*^				14.4%^**^			
RFL–Moral	Β	0.16		0.37^**^	−0.46^**^	0.30	0.55^*^	−0.31	−0.43^*^
	Δ*R*^2^ (12.4%)	23.6%^**^				4.4%			

*T*–*p* < 0.10;

* *p* < 0.05;

** *p* < 0.01.

High self-actualization as a meaning source predicted low RFL in all cases but one (the coping with problems reason).

For fear of social disapproval reason and, marginally significantly, for moral reasons, there were moderation effects: higher horizontal self-transcendence and lower self-actualization were related to these reasons only in the BE and NO groups, but not in the ID group. Lower coping with problems reason was predicted by higher well-being source of meaning in group BE (*β* = −0.32, *p* < 0.01, Δ*R*^2^ = 5.5%, *p* < 0.01). Probably patients with experience of self-destructive behavior were more tolerant to poor well-being, as Joiner (2005) suggests. Although this interpretation is close to the ideas of learned helplessness and stress vulnerability, it should be noted that none of these factors predicted self-destructive behavior so the underlying mechanism could be different from those typically described in that context.

Thus, lower self-actualization and higher horizontal self-transcendence were related to more reasons for living in general, specifically moral reasons and fear of social disapproval in the BE and NO groups but not in the ID group.

The fear of suicide reason was not related to sources of meaning.

### Relationships between sources of meaning and reasons for living after adjusting for clinical appraisals of the patients’ conditions

Only one major effect of clinical characteristics was significant: actual stress was related to a higher fear of suicide RFL in all three groups. Accordingly, we removed them from the analyses and stepwise added all possible moderators describing the interactions between the five clinical characteristics and the two dummy-coded groups (i.e., 10 interactions). If significant interaction effects were found, the relevant clinical characteristic was added at step 1 to check whether the moderation effect would remain.

As can be seen from Table 4, neither clinical characteristics nor group membership predicted the coping with problems, moral or fear of social disapproval reasons. However, in all the cases there was a moderation effect: in patients of the BE group suicidal readiness was related to lower coping with problems, fear, and moral reasons. The coping with problems reason was also related to a lower social support deficit in the NO group.

**Table 4:** Clinical group and characteristics and reasons for living: the results of moderation analysis.

Independent variables in moderation analysis		RFL–Coping with problems		RFL–Fear of social disapproval		RFL–Moral	
		Β	Δ*R*^2^	β	Δ*R*^2^	β	Δ*R*^2^
Step 1	BE group	−0.13	22.0%^*^	0.36	8.9%	0.24	7.8%
	NO group	0.16		0.37		0.13	
	Suicidal readiness	−0.19		−0.02		−0.24	
	Social support deficit	−0.22					
Step 2	In BE group–Suicidal readiness	−0.79^**^	17.7%^**^	−0.75^*^	10.2%^*^	−0.67^*^	7.9%^*^
	In NO group–Social support deficit	−0.49^*^					

* *p* < 0.05;

** *p* < 0.01.

It is interesting that no specific RFL were predicted by clinical characteristics.

The fear of suicide reason was higher in the BE group as compared to patients of the ID group (*β* = 0.35, *p* < 0.05, Δ*R*^2^ = 20.4%, *p* < 0.01) and NO group (*β* = −0.14, n.s.). This result was not surprising taking into account that the experience of suicide was much more real for people who made an attempt (BE group). In all three groups there was a major effect of actual stress (*β* = 0.26, *p* < 0.05, Δ*R*^2^ = 7.1%, *p* < 0.05): the higher the stress experienced by the patients, the higher was the fear of suicide RFL. This effect was not moderated by group.

The responsibility for their family and relationship with children reasons were unrelated to clinical characteristics in all the three groups.

Using a similar stepwise moderation analysis strategy, we tested whether meaningfulness remained a positive predictor for RFL in all the three groups after controlling for clinical characteristics. An additional aim was revealing possible interaction effects between group membership, clinical variables and meaningfulness. Only suicidal readiness and social support deficit were used in these analyses because they were associated with RFL and meaningfulness, as noted above.

After stepwise adjusting for group membership, suicidal readiness, social support deficit and their interactions (*R*^2^ = 26.3%, *p* < 0.01), meaningfulness still predicted coping with problems reason (baseline *R*^2^ = 41.1%, *p* < 0.01, after adding meaningfulness *β* = 0.52, *p* < 0.01, Δ*R*^2^ = 21.1%, *p* < 0.01), relationship with children reason (baseline *R*^2^ = 9.8%, *p* < 0.05, after adding meaningfulness *β* = 0.57, *p* < 0.01, Δ*R*^2^ = 27.4%, *p* < 0.01) and moral RFL (baseline *R*^2^ = 14.6%, *p* < 0.05, after adding meaningfulness *β* = 0.34, *p* < 0.05, Δ*R*^2^ = 10.8%, *p* < 0.05) but not for responsibility for family, fear of suicide or fear of social disapproval reasons where the effect of meaningfulness did not reach *p* < 0.05 significance level after adjusting for clinical variables.

## Discussion

*Psychological predictors for suicidal ideation and attempts among people in crisis life situations.* Differentiating between patients in crisis life situations who hold suicidal intentions and are at risk of suicide and those who are not is an important challenge. Although intuitively it seems obvious that suicidal ideation is a “first station” on the way to suicidal attempts, the approach of existential psychology suggests that suicidal ideation is not directly positively associated with the risk of suicidal behavior. As our data show, neither well- and ill-being measures (subjective happiness, subjective vitality, anxiety, depression), nor self-regulatory resources (action orientation, hardiness, coping strategies, tolerance for ambiguity), nor crisis of meaning and alienation differed between those patients who made suicidal attempts, those with suicidal ideation and those who came to crisis clinics by other reasons and who denied suicidal thoughts. Rather, the differences between these three groups referred to meaningfulness and reasons for living. This confirmed Hypothesis 1a and disconfirmed Hypotheses 1b and 1c.

It is interesting that reasons for living (especially the coping with problems reason) were higher in the NO group and hardly differed in the two other groups, while meaningfulness and some sources of meaning were equally high in the NO and ID groups and decreased in the BE group (Table 1). It looks as if the appearance of suicidal ideations covaried with a decrease in RFL and not in meaning, while the transition from suicidal ideation to suicidal attempts covaried with decreased meaningfulness (but not in increased alienation or crisis of meaning). It is a lowered positive meaning resource that seems to be most indicative of suicidal risk.

Clinically appraised suicidal readiness was higher in patients with suicidal attempts but did not significantly differ in the ID and NO groups (Table 1); it follows from this that in crisis situations suicidal ideation might not be indicative of suicidal risk. This partly confirmed Hypothesis 1. Moreover, patients with suicidal ideations were appraised by doctors as having the least social support deficit and marginally less affective symptoms.

Clear clinical “signs” of suicidal risk might be not very helpful in prevention because they are mostly based on already existing history of previous attempts, actual impulsivity and self-harming behavior. In other words, clinical appraisals seem to detect well suicidal risk in those who have already made suicidal attempts but fail to predict it in those who did not. In this context it is interesting that suicidal “readiness” was related to fewer reasons for living (especially coping with problems, moral reasons and fear of social disapproval) and lower meaningfulness, but in patients with suicidal attempts only.

*The relations among meaningfulness, crisis of meaning, and reasons for living.* While fewer reasons for living seem to be the major indicator of suicidal ideations and attempts, our further aim was revealing their psychological predictors. In line with Schnell’s theory, in all the three groups meaningfulness predicted more positive reasons for living. These effects were replicated using other measures of meaningfulness and alienation (Tables 2, 3 Hypothesis 2 is partly confirmed).

The hypothesis stating that the crisis of meaning is an independent predictor for coping with problems that could not be reduced to meaningfulness, was supported for the coping with problems reason only. Moreover, when we used other measures, alienation predicted the coping with problems RFL only in the BE group. In this group meaningfulness and crisis of meaning were just weakly correlated with each other, indicating that both feelings of meaningfulness and crisis of meaning may be high or low at the same time in these patients. No clear evidence for possible interaction between meaningfulness and crisis of meaning was found or replicated using other measure. Thus, the ‘disbalance’ between meaningfulness and crisis of meaning in patients with suicidal attempts was not related to reasons for living.

The relationship between meaningfulness and most reasons for living remained significant after controlling for clinically appraised suicidal “readiness” and a social support deficit (Table 4; the first part of Hypothesis 3 confirmed). There was no interaction between clinical appraisals and meaningfulness (the second part of Hypothesis 3 disconfirmed).

The data obtained below are in line with the existentialist statement that thinking about a suicide option is not a pathological symptom unless these thoughts are converted into actions or become obsessive. Awareness of suicide as an option is a sign of mature self-reflection, a precondition of viewing one’s life as an outcome of conscious choices rather than a succession of random events or fatal determinations. This self-reflection seems to be the key chain in this process which has no predetermined outcome. The impact of all the life circumstances, challenges, losses, traumas etc. is mediated by the personal meanings all these factors acquire in the context of the general life meaning.

It follows that prevention efforts are to be focused not at eliminating the factors “pushing” one to suicidal behavior, but rather at supporting inner strengths, conducive of a positive decision, to be. Clinicians could develop therapeutic strategies aimed at preventing suicidal thoughts and behaviors and improve the care management of suicidal patients through enhancing meaningfulness and reasons for living. Suicidal ideations might be thus transformed into saying “yes” to life and integrated on the existentialist basis into the positive vision of one’s life. Such efforts are being elaborated first of all within Logotherapy, Dialectical Behavior Therapy, and Cognitive Behavioral Therapies (see, e.g., Linehan, 2015; Bakhiyi et al., 2016).

## Conclusion

The data cast some light on the role of life meaning in suicide prevention. The role of meaningfulness as a buffering factor has received new support. Specifically, life meaningfulness predicted stronger reasons for living, while meaning deficit, or existential frustration (Frankl) seemed to be less critical. Qualitative characteristics of meaning in life in terms of its sources made some moderate contribution varying in the effect size. It follows that prevention efforts are to be focused not on eliminating the factors ‘pushing’ one to suicidal behavior, but rather on supporting inner strengths, conducive toward a positive decision, *to be*. A suicidal decision is made in a competition with the opposite option, saying Yes to one’s life. Our data support the belief that positive buffers, first of all meaningfulness of life and reasons for living influence this critical choice probably more than anything else (at least, we do not know of data showing that anything else is more important). Meaningfulness and reasons for living justify the choice in favor of life and empower one to reject the option of killing oneself.

### Limitations and perspectives

The data collection for this study was very complicated and took years. The results were encouraging; they seem to open interesting perspectives but hardly give definite answers. The key ideas underlying the study proved to be fruitful: the emphasis on meaning, the differentiation of positive and negative predictors (the former were stronger), the differentiation of suicidal behavior and mere ideation. Indeed, “though the physicality of death destroys man, the idea of death saves him” (Yalom, 1980, p.30).

The study had some important limitations. One is that we failed to use a follow-up assessment that would add much to our cross-sectional design. Secondly, although we used independent evaluation of the patients’ clinical status, these evaluations were made by clinical staff who already knew of the patient’s past suicide attempt history, and this knowledge likely contributed a substantial part of the variance of the clinical checklist scores.

New studies should address these limitations. A special task would be elaborating and testing interventions based on reflection upon the issue of suicide, as in psychotherapeutic approaches mentioned above.

## Data availability statement

The raw data supporting the conclusions of this article will be made available by the authors, without undue reservation.

## Ethics statement

Ethical review and approval was not required for the study on human participants in accordance with the local legislation and institutional requirements. The patients/participants provided their written informed consent to participate in this study.

## Author contributions

OK: data collection and research planning, DL: methodology, study design, and overall project administration, ER: controlled statistical analysis of the data, and OT was responsible for: literature overview and data administration. All authors contributed to the article and approved the submitted version.

## Funding

The study was supported by the HSE University Basic Research Program. The paper was prepared within the framework of the HSE University Basic Research Program.

## Conflict of interest

The authors declare that the research was conducted in the absence of any commercial or financial relationships that could be construed as a potential conflict of interest.

## Publisher’s note

All claims expressed in this article are solely those of the authors and do not necessarily represent those of their affiliated organizations, or those of the publisher, the editors and the reviewers. Any product that may be evaluated in this article, or claim that may be made by its manufacturer, is not guaranteed or endorsed by the publisher.

## Footnotes

^1^ https://www.einpresswire.com/article/457454620/american-association-of-suicidology-announces-aas365-campaign-for-national-suicide-prevention-month-and-beyond

^2^The diagnoses of the patients did not exclude their capability of giving informed consent personally.
